# The Terminal Oxidase Cytochrome *bd* Promotes Sulfide-resistant Bacterial Respiration and Growth

**DOI:** 10.1038/srep23788

**Published:** 2016-03-31

**Authors:** Elena Forte, Vitaliy B. Borisov, Micol Falabella, Henrique G. Colaço, Mariana Tinajero-Trejo, Robert K. Poole, João B. Vicente, Paolo Sarti, Alessandro Giuffrè

**Affiliations:** 1 Department of Biochemical Sciences and Istituto Pasteur- Fondazione Cenci Bolognetti, Sapienza University of Rome, Italy; 2 Belozersky Institute of Physico-Chemical Biology, Lomonosov Moscow State University, Leninskie Gory, Moscow 119991, Russian Federation; 3 Metabolism & Genetics Group, Research Institute for Medicines (iMed.ULisboa), Faculty of Pharmacy, University of Lisbon, Portugal; 4 Cell Biology Program, The Hospital for Sick Children, Toronto, ON, Canada; 5 Department of Molecular Biology and Biotechnology, The University of Sheffield, Sheffield S10 2TN, United Kingdom; 6 Instituto de Tecnologia Química e Biológica António Xavier, Universidade Nova de Lisboa, Oeiras, Portugal; 7 CNR Institute of Molecular Biology and Pathology, Rome, Italy

## Abstract

Hydrogen sulfide (H_2_S) impairs mitochondrial respiration by potently inhibiting the heme-copper cytochrome *c* oxidase. Since many prokaryotes, including *Escherichia (E.) coli*, generate H_2_S and encounter high H_2_S levels particularly in the human gut, herein we tested whether bacteria can sustain sulfide-resistant O_2_-dependent respiration. *E. coli* has three respiratory oxidases, the cyanide-sensitive heme-copper *bo*_3_ enzyme and two *bd* oxidases much less sensitive to cyanide. Working on the isolated enzymes, we found that, whereas the *bo*_3_ oxidase is inhibited by sulfide with half-maximal inhibitory concentration *IC*_50_ = 1.1 ± 0.1 μM, under identical experimental conditions both *bd* oxidases are insensitive to sulfide up to 58 μM. In *E. coli* respiratory mutants, both O_2_-consumption and aerobic growth proved to be severely impaired by sulfide when respiration was sustained by the *bo*_3_ oxidase alone, but unaffected by ≤200 μM sulfide when either *bd* enzyme acted as the only terminal oxidase. Accordingly, wild-type *E. coli* showed sulfide-insensitive respiration and growth under conditions favouring the expression of *bd* oxidases. In all tested conditions, cyanide mimicked the functional effect of sulfide on bacterial respiration. We conclude that *bd* oxidases promote sulfide-resistant O_2_-consumption and growth in *E. coli* and possibly other bacteria. The impact of this discovery is discussed.

Along with nitric oxide (NO) and carbon monoxide (CO), hydrogen sulfide (H_2_S) has been recognized as an important gaseous signalling molecule, playing a major role in human (patho)physiology^1^. Like NO and CO, H_2_S is a key regulator of many physiological processes in the cardiovascular, nervous, respiratory and gastrointestinal systems, among others. While exerting beneficial physiological effects at lower levels, at higher concentrations H_2_S can cause detrimental effects. In eukaryotes, depending on its concentration, H_2_S can have opposite effects on respiration (reviewed in^2^): at nanomolar concentrations it can sustain energy metabolism both as a substrate for the mitochondrial respiratory chain and as a vasodilator favouring O_2_ supply, whereas at higher levels it impairs cellular respiration via direct binding to and inhibition of mitochondrial cytochrome *c* oxidase (mtCcOX) (see^3^ and references therein). Sulfide inhibition of mtCcOX is very effective (*K*_i_ = 0.2–0.45 μM at pH = 7.4^3^,^4^), leading to dissipation of the mitochondrial membrane potential, consequent arrest of aerobic ATP production and eventually cell death^2^.

In mammalian tissues, H_2_S is enzymatically produced by cystathionine β-synthase (CBS), cystathionine γ-lyase (CSE) and via the combined action of 3-mercaptopyruvate sulfurtransferase (3-MST) and cysteine aminotransferase (reviewed in^5^). At variance from other compartments in the human body, in the intestinal lumen H_2_S is also generated by the gut microbiota through bacterial amino acid metabolism and *via* dissimilatory sulfate reduction by ‘sulfate-reducing bacteria’ (SRB)^6^. H_2_S levels in the gut are therefore high. Whereas the total sulfide pool content in the colon is around one millimolar^7^, the concentration of free H_2_S in the intestinal lumen was reported to be ca. 40–60 μM, as estimated by direct measurement of the gas in the rat cecum^8^,^9^ and analysis of human faecal samples^10^.

*E. coli* is a ubiquitous member of the human gut microbiota, with more than one strain commonly colonizing the large intestine at the same time. Since *E. coli*, like the other microorganisms inhabiting the gut, lives in a particularly H_2_S-enriched microaerobic niche, the question arises as to whether this microorganism can accomplish O_2_-dependent respiration without being inhibited by H_2_S. The *E. coli* respiratory chain possesses three terminal oxygen reductases, utilizing quinols as reducing substrates: the cyanide-sensitive cytochrome *bo*_3_ enzyme and the *bd*-I and *bd*-II oxidases, much less sensitive to cyanide^11^,^12^. Cytochrome *bo*_3_ belongs to the superfamily of heme–copper oxygen reductases that includes mtCcOX. The enzyme contains three redox-active metal centres: the low-spin heme *b* involved in quinol oxidation and a binuclear site composed of heme *o*_3_ and Cu_B_, where O_2_ reduction to water takes place. On the contrary, *bd*-I and *bd*-II are cytochrome *bd*-type O_2_-reductases phylogenetically unrelated to heme–copper oxidases^12^. They have no copper, but contain three hemes: the low-spin heme *b*_558_ (the primary electron acceptor from the quinol), and the two high-spin hemes *b*_595_ and *d* (possibly forming a di-heme site for O_2_ reduction, see^12^ and references therein). Cytochrome *bo*_3_ predominates in *E. coli* under high aeration, whereas O_2_-limiting conditions such as those found in the human gut stimulate the expression of the cytochromes *bd*-I and *bd*-II^13^,^14^,^15^. The three *E. coli* terminal oxidases all generate a proton motive force, but cytochrome *bo*_3_ is the only one able to pump protons, thus being twice as effective as *bd*-type cytochromes in terms of energy transduction^16^. Besides its role in bacterial energy metabolism, cytochrome *bd*-I was suggested to serve other physiological functions, being implicated in the bacterial response to oxidative and nitrosative stress^17^,^18^,^19^,^20^.

In this work, we examined the effect of sulfide on the O_2_ reductase activity of the three terminal oxidases of *E. coli* and tested the ability of these enzymes to sustain bacterial growth and O_2_ consumption in the presence of sulfide.

## Results

### Effect of NaHS on isolated *E. coli* terminal oxidases

**Figure 1:**
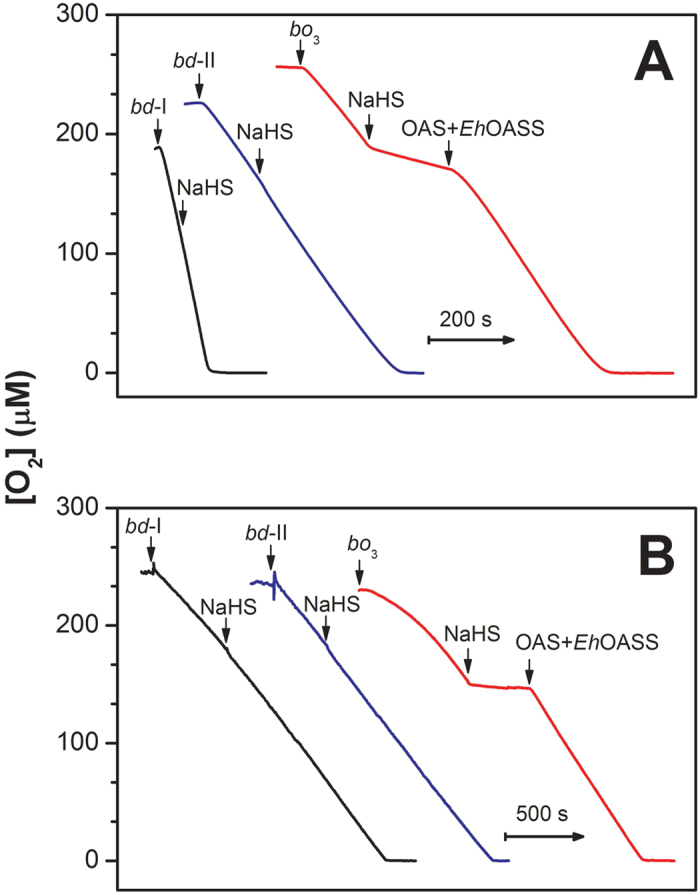
Effect of NaHS on *E. coli* terminal oxidases. (**A**) O_2_ reductase activity of the isolated cytochromes *bd*-I (20 nM), *bd*-II (2.5 nM) and *bo*_3_ (6 nM) as measured at 25 °C in the presence of DTT (10 mM) and Q_1_ (0.25 mM). O_2_ consumption rates measured prior to NaHS addition (mean ± standard deviation, n = 3): 1.62 ± 0.07 μM O_2_/s (*bd*-I); 0.54 ± 0.02 μM O_2_/s (*bd*-II) and 0.46 ± 0.02 μM O_2_/s (*bo*_3_). O_2_ consumption by cytochrome *bo*_3_ is rapidly inhibited (~85%) by 7.2 μM NaHS, to be quickly and completely restored upon removal of sulfide from solution following the addition of 200 μM OAS and 216 nM *Eh*OASS. On the contrary, NaHS (58 μM) does not affect the oxidase activity of either cytochrome *bd*-I or cytochrome *bd*-II. (**B**) O_2_ consumption by cell suspensions of the mutant strains expressing cytochrome *bd*-I (400 μl cells with OD_600_ = 1.85), cytochrome *bd*-II (600 μl cells with OD_600_ = 1.17) or cytochrome *bo*_3_ (200 μl cells with OD_600_ = 2.45), as the only terminal oxidase. O_2_ consumption rates measured prior to NaHS addition (mean ± standard deviation, n = 3): 0.20 ± 0.07 μM O_2_/s (*bd*-I); 0.18 ± 0.02 μM O_2_/s (*bd*-II) and 0.19 ± 0.02 μM O_2_/s (*bo*_3_). When sustained solely by cytochrome *bo*_3_, cell respiration is rapidly inhibited by 50 μM NaHS, to be quickly and completely restored following sulfide removal on addition of OAS (200 μM) and *Eh*OASS (216 nM). No inhibition is observed following the addition of NaHS (50 μM), when respiration is sustained by the only cytochrome *bd*-I or *bd*-II.

**Figure 2:**
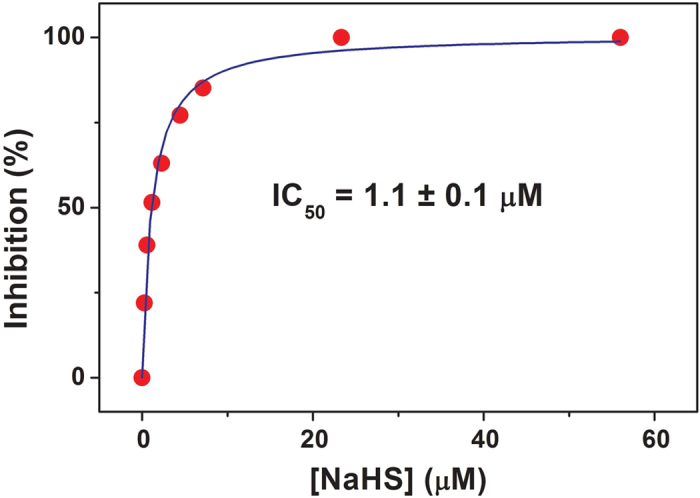
NaHS inhibition of isolated cytochrome *bo*_3_. Percentage inhibition of the O_2_ reductase activity of isolated cytochrome *bo*_3_ (6 nM) measured at increasing concentration of NaHS, in the presence of the 10 mM DTT and 0.25 mM Q_1_.

The effect of sulfide on the O_2_ reductase activity of the *E. coli* respiratory oxidases, cytochromes *bo*_3_, *bd*-I and *bd*-II, was initially investigated testing the ability of each purified oxidase to consume O_2_ before and after addition of the sulfide donor NaHS. In these assays, O_2_ consumption was measured in the presence of dithiothreitol (DTT) and 2,3-dimethoxy-5-methyl-6-(3-methyl-2-butenyl)-1,4-benzoquinone (Q_1_) as the reducing system. As shown in Fig. 1A, NaHS (~7 μM) rapidly and effectively inhibits the O_2_ reductase activity of the isolated cytochrome *bo*_3_. The enzyme is inhibited with an apparent half-maximal inhibitory concentration *IC*_50_ = 1.1 ± 0.1 μM (Fig. 2). The inhibition of cytochrome *bo*_3_ is fully reversible. A rapid and complete recovery of the O_2_ reductase activity of the isolated enzyme was observed, when sulfide was quickly removed from solution by addition of an excess of *O*-acetyl-L-serine (OAS) and catalytic amounts of the sulfide-consuming *O*-acetylserine sulfhydrylase enzyme from *Entamoeba histolytica* (*Eh*OASS, Fig. 1A). Sulfide consumption by *Eh*OASS in the presence of OAS was assessed independently using a H_2_S-selective electrode (Figure S1). Notably, while being an effective inhibitor of *E. coli* cytochrome *bo*_3_, NaHS proved to be unable to inhibit the two *E. coli bd*-type oxidases. Addition of NaHS, even at high concentration (58 μM), did not alter the O_2_ consumption catalyzed by the *bd*-I or *bd*-II enzyme in the presence of DTT and Q_1_ (Fig. 1A). No O_2_ consumption stimulation by the OAS/*Eh*OASS sulfide-scavenging system was observed in control oxygraphic experiments carried out in the absence or presence of the isolated oxidases (not shown).

### Effect of NaHS on *E. coli* respiration

**Figure 3:**
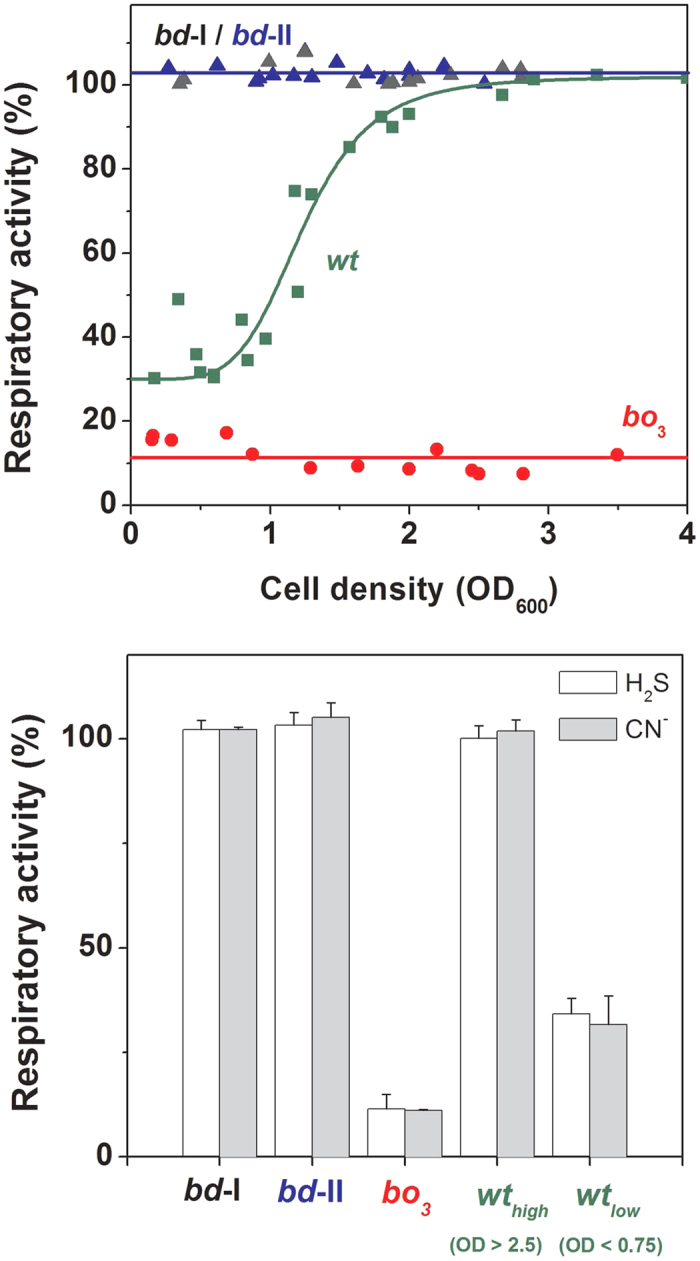
Effect of NaHS and cyanide on respiration of *E. coli* cells. (**Top**) Residual respiratory activity measured after the addition of 50 μM NaHS to *E. coli* cells collected at the reported cell density. (**Bottom**) Comparison of the effect of cyanide and sulfide on cell respiration: respiratory activity measured after the addition of 50 μM NaHS or 50 μM NaCN to wild-type and mutant *E. coli* cells. Data (mean ± standard deviation) refer to the control activity measured before the addition of inhibitors (taken as 100%).

The striking results obtained with the isolated enzymes prompted us to explore the effect of sulfide on *E. coli* cell respiration. To this end, we investigated aerobic cultures of *E. coli* (see Methods for details) and tested the effect of NaHS on cell respiration along cell growth, i.e., at increasing cell density. We initially assayed three mutant strains each expressing a single terminal oxidase (*bo*_3_, *bd*-I or *bd*-II). The results were remarkably similar to those obtained with the isolated enzymes. O_2_ consumption by *E. coli* cells expressing solely cytochrome *bo*_3_ was quickly and fully inhibited upon addition of 50 μM NaHS (Fig. 1B). As observed with the isolated *bo*_3_ enzyme, the inhibition was promptly and fully restored upon sulfide depletion by the *Eh*OASS/OAS system (Fig. 1B). In contrast, no inhibition was observed following the addition of 50 μM NaHS to *E. coli* cells expressing either *bd*-I or *bd*-II as the only terminal oxidase (Fig. 1B). The results on the three mutant strains proved to be independent of the density at which cells were collected and assayed (Fig. 3, top panel). Similarly to NaHS, cyanide (50 μM) almost completely abolished O_2_-consumption in *E. coli* cells expressing only the *bo*_3_ oxidase, whereas it was essentially ineffective when respiration was sustained by either *bd* oxidase (Fig. 3, bottom panel).

The effect of NaHS on respiration of the wild-type strain was assessed in the same way. Namely, we investigated aerobic cultures in which a change in oxidase expression from cytochrome *bo*_*3*_ to the cytochromes of the *bd-*type is expected to take place along cell growth, following a progressive reduction in O_2_ availability in the medium^21^,^22^. Accordingly, when cells were assayed in an early phase of the culture (OD_600_ < 0.7), most of respiration (65–70%) proved to be sensitive to NaHS or cyanide (both at 50 μM, Fig. 3). In contrast, with cell growth bacterial O_2_-consumption became progressively less sensitive to sulfide inhibition and, in a late phase of the culture (OD_600_ > 2.5), NaHS or cyanide caused only marginal effects on respiration (Fig. 3).

Altogether these results show that, unlike the heme-copper *bo*_*3*_ oxidase, *E. coli bd* oxidases enable O_2_-dependent respiration in the presence of sulfide.

### Effect of NaHS on *E. coli* cell growth

**Figure 4:**
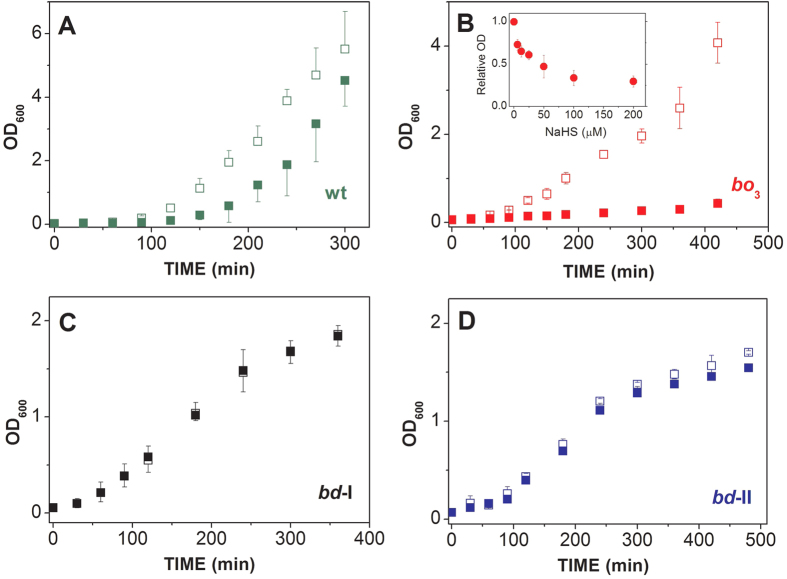
Effect of NaHS on *E. coli* cell growth. Cell growth of *E. coli* wild-type (**A**) and mutant strains with *bo*_3_ (**B**), *bd-I* (**C**) or *bd-II* (**D**) as the only terminal oxidase, assayed in the presence (‘closed symbols’) or absence (‘open symbols’) of 200 μM NaHS. *Inset to panel B*: Effect of NaHS on the growth of the *bo*_*3*_-only expressing mutant, as evaluated at 2 hours after addition of NaHS used at the indicated concentrations. ‘Relative OD’ indicates the ratio between the optical density measured at 600 nm in the presence of NaHS and the one recorded after the same period of time (2 hours) in the absence of NaHS. Data expressed as mean ± standard deviation.

The lack of sulfide inhibition of cytochromes *bd*-I and *bd*-II, as opposed to the high sensitivity displayed by the *bo*_3_ oxidase, prompted us to test whether the *bd*-type oxidases, besides enabling respiration, promote *E. coli* cell growth in the presence of sulfide. We investigated the effect of sulfide on the growth of both the wild-type and the three respiratory mutant strains. Following the addition of 200 μM NaHS, the wild-type strain showed a delayed growth (Fig. 4A), while the growth of the *bo*_3_-expressing strain was severely impaired (Fig. 4B). Lacking *bd* oxidases, the latter strain proved to be highly sensitive to sulfide, with 6 μM NaHS causing ~25% reduced cell growth, as evaluated at 2 hours after NaHS addition (inset Fig. 4B). In contrast, no or very little effect on cell growth was observed over the same time window after addition of 200 μM NaHS to the strains expressing either *bd*-I or *bd*-II as the only terminal oxidase (Fig. 4C,D). Altogether, these data show that, unlike the *bo*_3_ oxidase, the cytochromes *bd*-I and *bd*-II sustain *E. coli* growth in the presence of sulfide.

## Discussion

Together with NO and CO, H_2_S is presently considered a highly relevant signalling molecule in human (patho)physiology. It has long been recognized that many prokaryotes, including the model organism *E. coli* and numerous other members of the human gut microbiota, generate H_2_S (see^6^ and references therein). Bacteria can accomplish H_2_S production by several pathways, including cysteine degradation by L-cysteine desulfhydrase, and dissimilatory sulfate reduction by SRB (see^6^ and references therein). In a recent study, it was reported that orthologs of the mammalian H_2_S-synthesizyng enzymes CBS, CSE and 3-MST are widespread in the bacterial world and contribute to H_2_S generation, as demonstrated for several bacteria by genetic manipulation^23^. As an example, *E. coli* was shown to harbour an ortholog of 3-MST significantly contributing to bacterial H_2_S synthesis. Notably, in the same study H_2_S production was shown to enhance antibiotic resistance in all tested bacteria, thereby providing an adaptive advantage.

The presence of numerous H_2_S-producing bacteria in the human gut makes this compartment particularly enriched in H_2_S compared to other tissues, with the free gas reaching in the intestinal lumen concentrations as high as 40–60 μM^8^,^9^,^10^. Relevant to human (patho)physiology, bacteria-derived H_2_S is emerging as a key regulator of several physiological functions not only in the gastrointestinal system, but also throughout the human body^1^. Moreover, it has been recently suggested that the differential susceptibility of mutualistic microbes to sulfide toxicity may contribute to shape the human gut microbiota^6^, a recognized factor contributing to human health and disease. In turn, the host H_2_S systemic bioavailability and metabolism have been found to be profoundly affected by the microbiota in studies on germ-free mice^24^. Altogether these observations provide evidence for interplay between H_2_S and the human microbiota, with important consequences on human health.

Though currently considered a key signalling molecule, H_2_S has long been known as a mere poison. Toxicity has been related to the ability of H_2_S to bind heme proteins and inhibit cellular respiration targeting mtCcOX (see^3^ and references therein). Indeed, H_2_S is a potent (*K*_i_ = 0.2–0.45 μM^3^,^4^), non-competitive inhibitor of this respiratory enzyme, the inhibition being reversible, independent of oxygen concentration^25^, but dependent on pH^26^. Sulfide inhibition of isolated mtCcOX in turnover with ascorbate and cytochrome *c* is relatively fast, occurring at an initial rate constant of 2.2 × 10^4^ M^−1^ s^−1^, as measured at pH 7.4 3. The inhibited enzyme exhibits sulfide bound to ferric heme *a*_3_^27^,^28^, with Cu_B_ in the cuprous state possibly bound to a second H_2_S molecule, as revealed by electron paramagnetic resonance (EPR) spectroscopy^29^. The mechanism of inhibition of mtCcOX is only partly understood, yet the reaction was suggested to involve the binding of H_2_S to the enzyme in turnover at cupric or cuprous Cu_B_, followed by intramolecular transfer of H_2_S to ferric heme *a*_3_, eventually blocking the reaction with O_2_^3^.

The well-known toxicity of H_2_S on mitochondrial respiration prompted us to address whether bacterial O_2_-dependent respiration can be accomplished in a H_2_S-enriched environment such as the human gut, thereby providing an adaptive advantage in terms of bacterial growth. This issue was addressed in the present study working on the model organism *E. coli*, a ubiquitous member of the human gut microbiota. Namely, we investigated the effect of sulfide on the O_2_ reductase activity of each of the three terminal respiratory oxidases of this bacterium (cytochromes *bo*_3_, *bd*-I and *bd*-II), and tested the ability of these enzymes to sustain O_2_ consumption and bacterial cell growth in the presence of sulfide. Using NaHS as a H_2_S donor, we carried out experiments on the isolated enzymes, as well as on the wild-type and three respiratory mutant *E. coli* strains each expressing only a single terminal oxidase. NaHS is commonly used as a donor of the cell permeant H_2_S, because in aqueous solution HS^−^ equilibrates with H_2_S and S^2−^, according to the p*K*_a1_ ~7.0 (H_2_S/HS^−^) and p*K*_a2_ ~19 (HS^−^/S^2−^) measured at 25 °C. At pH = 7.0–7.4, ~30–50% of HS^−^ is thus expected to be protonated to H_2_S, with S^2−^ being present in negligible amounts.

As a new finding we report that, whereas the heme-copper *bo*_3_ oxidase is highly sensitive to sulfide inhibition (*IC*_50_ =1.1 ± 0.1 μM, Figs 1 and 2), the two *bd* oxidases (*bd*-I and *bd*-II) are remarkably insensitive to sulfide (Fig. 1), as confirmed by measuring the effect of NaHS on O_2_ consumption by the purified terminal oxidases (Fig. 1A) or by whole cells (Figs 1B and 3). In agreement with these finding, cell growth proved to be severely impaired by sulfide in an *E. coli* mutant strain expressing only the *bo*_3_ oxidase (Fig. 4B), but unaffected in mutant strains expressing either *bd*-I or *bd*-II as the only terminal oxidase (Fig. 4, panel C,D). Consistently, in the wild-type strain, H_2_S affected cell growth and respiration only in the early phase of the culture, when O_2_ availability is expected to be still sufficiently high to favour the expression of the *bo*_3_ oxidase, but it caused no effect in a late phase of the culture, when O_2_ limitation is expected to stimulate the expression of *bd* oxidases (Fig. 4A).

Altogether, these observations led us to conclude that, at variance with the heme-copper *bo*_3_ oxidase that is potently and reversibly inhibited by sulfide, both *E. coli bd* oxidases are sulfide-insensitive and thus able to sustain cell respiration and growth in the presence of considerably high levels of sulfide. Although the molecular basis for the remarkable sulfide insensitivity of the *E. coli bd* oxidases remains to be elucidated, it may originate from the lack of Cu_B_, which was indeed suggested to be implicated in sulfide inhibition of mtCcOX^3^. In this regard, still possibly due to the lack of Cu_B_, it is noteworthy that *bd* oxidases are not only more resistant to NO inhibition than heme-copper oxidases^30^,^31^,^32^, but also poorly sensitive to other commonly used oxidase inhibitors, such as cyanide^12^ and azide^33^. On this basis, cyanide and sulfide are expected to exert similar inhibitory effects on *E. coli* respiration, as observed in the present study (Fig. 3).

As shown here for *E. coli*, it is likely that *bd* oxidases confer sulfide resistance also to other microorganisms. The *bd* oxidases are indeed widespread in the prokaryotic world and have been identified in numerous enterobacteria^34^, where expression of these oxidases is likely stimulated in the microaerobic conditions found in the human colon. In view of the novel results presented here, it will be important to test whether *bd* oxidases, by conferring sulfide resistance, play a role in shaping the human gut microbiota, thereby impacting human (patho)physiology. Furthermore, based on these data, *bd* oxidases may represent very attractive targets for the development of next-generation antimicrobials against pathogenic enterobacteria^18^,^20^,^35^. Finally, the finding that *bd* oxidases enhance bacterial resistance to sulfide, if representing a hallmark of this protein family, may pave the way to biotechnological applications aimed at increasing bacterial sulfide resistance.

## Methods

### Materials, bacterial strains and growth conditions

All chemicals were purchased from Sigma unless otherwise indicated. NaHS stock solutions were prepared by dissolving NaHS in degassed water or phosphate buffer saline, and the overall concentration of sulfide species (H_2_S/HS^−^/S^2−^) in solution was determined spectrophotometrically according to^36^. All *E. coli* strains used were K-12 derivatives; MG1655 (RKP5416) was the wild type^37^ from which the respiratory mutants, TBE025 (MG1655 Δ*cydB nuoB appB::kan*), TBE026 (MG1655 Δ*cydB nuoB cyoB::kan*) and TBE037 (MG1655 Δ*appB nuoB cyoB::kan*) were derived, respectively expressing cytochrome *bo*_3_, *bd*-II and *bd*-I as the only terminal oxidase (mutants kindly given by Alex Ter Beek and Joost Teixeira de Mattos, University of Amsterdam). These strains carry the same mutant alleles as described by Bekker *et al*.^38^. *E. coli* cells were grown in 50 mL-Falcon tubes, in 5 mL Luria Bertani (LB) medium supplemented with 30 μg/mL kanamycin, at 37 °C and 200 rpm. For growth studies, cells were grown as described above in the absence or presence of NaHS (6–200 μM) added to cells at an OD_600_ of about 0.05.

### Purification of terminal oxidases from *E. coli*

The cytochromes *bd*-I, *bd*-II and *bo*_3_ were isolated from the *E. coli* strains GO105/pTK1, MB37 and GO105/pJRhisA, respectively, as previously described^39^,^40^,^41^. The concentration of the cytochromes *bd*-I and *bd*-II was determined from the difference absorption spectrum using Δ*ε*_628-607_ = 10.8 mM^−1^ cm^−1^ for the dithionite-reduced *minus* ‘as prepared’ proteins. Cytochrome *bo*_3_ concentration was estimated from the Soret absorption band of the oxidized enzyme using ε_407_ = 183 mM^−1^ cm^−1^. UV-visible absorption spectra were acquired in an Agilent Cary 60 spectrophotometer.

### Purification and H_2_S consumption by recombinant *O*-acetylserine sulfhydrylase from *Entamoeba histolytica*

The *O*-acetylserine sulfhydrylase-encoding gene (*Eh*OASS, Genbank XM_643199.1) was PCR-amplified from *Entamoeba histolytica* HM-1:IMSS genomic DNA using the forward primer *5*′-CATATGATGGAACAAATAAGTATTAGC and the reverse primer *5*′-AACGTTTTATTCATTCAATAATGAATCAAG, containing the NdeI and HindIII restriction sites respectively. The PCR product was cloned into the Topo TA pCR2.1 vector, digested with the NdeI and HindIII restriction enzymes, and gel purified. The DNA insert was subcloned into the NdeI and HindIII restriction sites of the pET28b expression vector, yielding the pET-*Eh*OASS construct encoding N-terminally 6xHis-tagged *Eh*OASS. pET-*Eh*OASS was used to transform *E. coli* BL21 (DE3). Cells were grown at 37 °C in LB broth supplemented with 25 mg/L kanamycin (Nzytech) until OD_600_ reached 0.4–0.5. *Eh*OASS expression was induced with 0.1 mM isopropyl-β-D-thiogalactoside addition and the cultures moved to 30 °C, 130 rpm for 4 h. Cells were harvested and the pellet resuspended in 10 mL/L culture of buffer A (50 mM potassium phosphate, 300 mM KCl, pH 7.5, 10% glycerol) containing 1 mg/mL lysozyme, 1 mM phenylmethylsulfonyl fluoride and deoxyribonuclease I. After 30-min incubation on ice, cells were disrupted by sonication, centrifuged at 8200 g (5 min, 4 °C) and imidazole was added to the supernatant to a final concentration of 10 mM. Protein purification steps were performed in an Åkta Prime (GE Healthcare) chromatography system. Affinity purification of the His-tagged protein was performed using a HisTrap FF crude 1-mL column previously equilibrated with buffer A containing 10 mM imidazole (buffer B). The cleared supernatant was loaded onto the column at 1 mL/min and the column was washed with 25 column volumes of buffer B followed by a linear gradient of 15 column volumes up to 500 mM imidazole. Pooled protein fractions were loaded onto a PD10 (GE Healthcare) desalting column for imidazole removal, equilibrated and washed with buffer A. *Eh*OASS-containing fractions were concentrated with Amicon Ultra-15 centrifugal filter units (30 kDa cut-off) and loaded onto a size-exclusion 120-ml Superdex S-200 (GE Healthcare) column, equilibrated and eluted with buffer A at 0.7 mL/min. *Eh*OASS fractions were pooled; protein purity was assessed by SDS-PAGE and protein concentration was determined by the Bradford assay. As previously reported^42^, pure *Eh*OASS eluted as a dimer of ~38 kDa monomers (Figure S1).

H_2_S consumption by *Eh*OASS was measured at 20 °C in 100 mM HEPES, 260 U/mL catalase, 100 μM EDTA pH 7.0, using an ISO-H2S-2 hydrogen sulfide sensor coupled to an Apollo 4000 Free Radical Analyzer (World Precision Instruments). In these assays the concentration of H_2_S in solution was obtained from the nominal concentration of the NaHS added, assuming 1:1 partition between HS^-^ and H_2_S at pH 7.0, according to the pK_a_ of H_2_S.

### O_2_ consumption measurements

Oxygraphic measurements were carried out at 25 °C in 100 mM Na/phosphate pH 7.4, using a high-resolution respirometer (Oxygraph-2k, Oroboros Instruments) with a 1.5 mL chamber. The buffer was supplemented with 0.1 mM EDTA and either 0.05% *N*-lauroyl-sarcosine (cytochrome *bd*-I) or 0.02% dodecyl-β-D-maltoside (cytochrome *bd*-II and cytochrome *bo*_3_) in the assays on isolated oxidases. The apparent *IC*_50_ of NaHS for the O_2_-reductase activity of the isolated *bo*_3_ oxidase was obtained by plotting the percentage inhibition of the enzyme as a function of NaHS concentration and fitting the data to the Hill equation^43^, assuming a Hill coefficient *n* = 1.

## Additional Information

**How to cite this article**: Forte, E. *et al*. The Terminal Oxidase Cytochrome *bd* Promotes Sulfide-resistant Bacterial Respiration and Growth. *Sci. Rep.* **6**, 23788; doi: 10.1038/srep23788 (2016).

## Supplementary Material

Supplementary Information
