# Supplementary material for: The Terminal Oxidase Cytochrome bd Promotes Sulfide-resistant Bacterial Respiration and Growth
Source: Sci Rep. 2016 Mar 31;6:23788. doi: 10.1038/srep23788 (PMC4815019; doi:10.1038/srep23788)
Supplement: Supplementary Information [file srep23788-s1.pdf]

SUPPLEMENTARY INFORMATION

"The Terminal Oxidase Cytochrome *bd* Promotes Sulfide-resistant Bacterial Respiration and Growth"

**Elena Forte, Vitaliy B. Borisov, Micol Falabella, Henrique G. Colaço,
Mariana Tinajero-Trejo, Robert K. Poole, João B. Vicente, Paolo Sarti, Alessandro Giuffrè**

Figure S1: H_2S consumption by the *Entamoeba histolytica O*-acetylserine sulfhydrylase (*Eh*OASS)

(A) purified recombinant *Eh*OASS elutes as dimer of ~38 kDa monomers , as judged by size-exclusion chromatography. (B) amperometric measurement of H_2S consumption by *Eh*OASS. T = 20 °C. Buffer: 100 mM HEPES pH 7.0 containing 260 U/mL catalase and 100 μM EDTA. After three sequential additions of 0.75 μM H_2S, *O*-acetylserine (100 μM) was added and the reaction was initiated by addition of *Eh*OASS (9 nM). Under the tested conditions, *Eh*OASS efficiently consumes H_2S with an apparent turnover number of 35 s^{-1}.

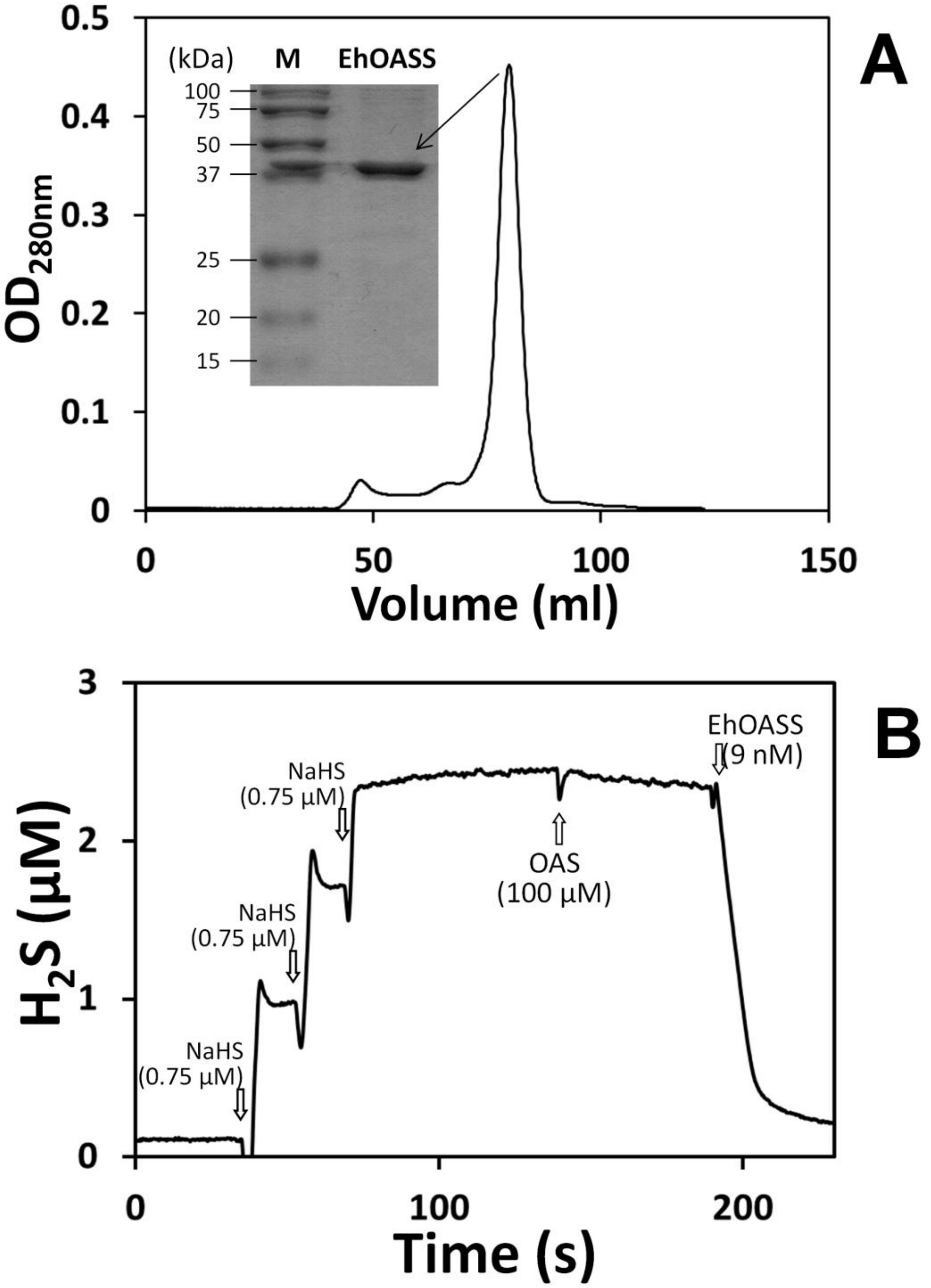

A
(kDa)
M
EhOASS
100
75
50
37
25
20
15
0.5
0.4
0.3
0.2
0.1
0
OD280nm
0
50
100
150
Volume (ml)
B
3
2
1
0
H2S (µM)
NaHS
(0.75 µM)
NaHS
(0.75 µM)
NaHS
(0.75 µM)
OAS
(100 µM)
EhOASS
(9 nM)
0
100
200
Time (s)

Figure S1
